# Patterns and correlates of physical activity: a cross-sectional study in urban Chinese women

**DOI:** 10.1186/1471-2458-7-213

**Published:** 2007-08-21

**Authors:** Adriana L Jurj, Wanqing Wen, Yu-Tang Gao, Charles E Matthews, Gong Yang, Hong-Lan Li, Wei Zheng, Xiao-Ou Shu

**Affiliations:** 1 Department of Medicine, Vanderbilt Epidemiology Center and Vanderbilt-Ingram Cancer Center, Vanderbilt University Medical Center, Nashville, TN 37203, USA; 2 Currently at the South Carolina Cancer Prevention and Control Program, South Carolina Public Health Consortium, University of South Carolina, Columbia, SC 29208, USA; 3 Department of Epidemiology, Shanghai Cancer Institute, Shanghai, PR China

## Abstract

**Background:**

Inactivity is a modifiable risk factor for many diseases. Rapid economic development in China has been associated with changes in lifestyle, including physical activity. The purpose of this study was to investigate the patterns and correlates of physical activity in middle-aged and elderly women from urban Shanghai.

**Methods:**

Study population consisted of 74,942 Chinese women, 40–70 years of age, participating in the baseline survey of the Shanghai Women's Health Study (1997–2000), an ongoing population-based cohort study. A validated, interviewer-administered physical activity questionnaire was used to collect information about several physical activity domains (exercise/sports, walking and cycling for transportation, housework). Correlations between physical activity domains were evaluated by Spearman rank-correlation coefficients. Associations between physical activity and socio-demographic and lifestyle factors were evaluated by odds ratios derived from logistic regression.

**Results:**

While more than a third of study participants engaged in regular exercise, this form of activity contributed only about 10% to daily non-occupational energy expenditure. About two-thirds of women met current recommendations for lifestyle activity. Age was positively associated with participation in exercise/sports and housework. Dietary energy intake was positively associated with all physical activity domains. High socioeconomic status, unemployment (including retirement), history of chronic disease, small household, non-smoking status, alcohol and tea consumption, and ginseng intake were all positively associated with exercise participation. High socioeconomic status and small household were inversely associated with non-exercise activities.

**Conclusion:**

This study demonstrates that physical activity domains other than sports and exercise are important contributors to total energy expenditure in women. Correlates of physical activity are domain-specific. These findings provide important information for research on the health benefits of physical activity and have public health implications for designing interventions to promote participation in physical activity.

## Background

Substantial data from the literature indicates that physical activity is an important modifiable risk factor for many diseases. Regular physical activity has been linked to a reduced risk of coronary heart disease [1,2], hypertension [3,4], stroke [5,6], type 2 diabetes mellitus [7,8], certain cancers [9-12], osteoporosis [13], and obesity [14-16], as well as decreased cardiovascular and overall mortality [17-19]. According to the World Health Organization (WHO), physical inactivity is responsible for 1.9 million deaths globally every year [20]. Despite this body of knowledge and sustained efforts to promote increased physical activity participation, the prevalence of physical activity in most industrialized countries remains low [21-24]. Women have been consistently found to have lower rates of participation in physical activity than men [21,22,24-28], although most of these studies assessed leisure-time physical activity only [22,25-27]. As physical activity occurs in multiple social aspects, other domains of physical activity are particularly important contributors to energy expenditure for women because they tend to spend more time than men participating in housekeeping, shopping, and food preparation activities [29]. The amount of energy spent in activities related to the household was higher for women than for men (33.3% vs. 20.1%), and leisure-time physical activity contributed only 5% of the total daily energy expenditure in a study carried out in a sample of US adults by Dong et al. [30].

Successful development and implementation of interventions to increase participation in physical activity can benefit from thorough understanding of factors associated with this behavior. A large body of research reviewed by Trost and colleagues has identified various personal, social, and environmental correlates of physical activity [31]. Younger age [25,26,32], higher level of education [22,26,32-36], and higher income [34-37] are well-documented factors associated with increased participation in physical activity. Leisure-time physical activity was also associated with alcohol consumption [33,38] and non-smoking status [26,27,34,35,37], as well as with low intake of fat and high intake of fruits and vegetables [37-39]. However, little is known about correlates of other domains of physical activity such as housework or commuting, which have been found to have health benefits [6,11,40].

Rapid economic development in China has been associated with an increased prevalence of overweight and several chronic diseases [41-43], paralleling changes in lifestyle, including physical activity. While data regarding prevalence and correlates of physical activity in China are not well described, the Shanghai Women's Health Study (SWHS), a population-based cohort study, represents a unique resource for characterizing physical activity patterns and their effect on various health outcomes.

The purpose of this study was to describe the patterns of, and interrelations between, physical activity domains, including exercise/sports, transportation, and housework. The study also examines the associations of physical activity domains with socio-demographic and lifestyle factors, and with health status in a population of middle-aged and older Chinese women using the baseline survey data of the SWHS.

## Methods

### Study population

The authors examined baseline data from the SWHS, an ongoing population-based prospective cohort study. The study was approved by the Institutional Review Boards of all participating institutions, and written informed consent was obtained from all participants. A detailed description of the study methodology has been published elsewhere [44]. Briefly, eligible participants for the SWHS were all women 40–70 years of age, residing in seven geographically defined areas of urban Shanghai, China. The study communities were selected because they were similar to urban Shanghai in terms of disease incidence rates and demographic characteristics. Before the study was initiated, rosters of all female residents between the ages of 40 and 70 were obtained from administrative offices of the study communities. All eligible women were approached in their homes by a local community health worker and a trained interviewer to determine interest in participating in the study. After obtaining informed consent from participants, a second home visit was scheduled to collect the self-administered questionnaire provided at the first visit, and to conduct an in-person interview, take anthropometric measurements and collect biological samples.

Of 81,170 eligible participants, 75,221 (92.7%) completed the baseline survey between 1997 and 2000. Reasons for nonparticipation included: refusal (n = 2,407, 3.0%), absence during the recruitment period (n = 2,073, 2.6%), and other miscellaneous reasons (n = 1,469, 1.8%). Completion of the self-administered questionnaire identified 279 women (0.4%) younger than 40 or older than 70 years that were further excluded, leaving 74,942 women in the baseline cohort.

### Assessment of physical activity

Physical activity data were collected via an in-person interview using a physical activity questionnaire (PAQ) that was validated in a sample of approximately 200 women from the SWHS [45]. Reproducibility (2-year test-retest) for adolescent and adult exercise participation (k = 0.85 and k = 0.64, respectively), and adult exercise energy expenditure (ICC = 0.70) were reasonable. Validity evaluated by comparing the results of the first and second administrations of the PAQ with the 7-day physical activity logs and the 7-day physical activity recalls was also good; moderate to strong correlations were found for most physical activity variables (Spearman correlation coefficients between 0.33–0.87) [45].

At the baseline survey, participants were first asked if they had engaged in regular exercise/sports (at least once a week for at least three months a year) during the preceding five years. Exercisers were asked to report details for up to three types of exercise/sports (i.e. type, hours/week, and years of participation in each activity), as well as how often they had sweated during exercise. Likewise, they were asked whether they had engaged in regular exercise during adolescence (13–19 years of age), the average duration (hours/week), and length of participation (years). Participation in athletic teams during adolescence was also evaluated. In terms of non-exercise activities, women were asked about participation in active transportation (i.e. walking and cycling to/from work, daily errands), stair climbing (flights of stairs/day), and housework (hours/day). Finally, women were asked to report the proportion of housework that they did by themselves. The time frame for these non-exercise activities was the preceding year.

Physical activity energy expenditure was estimated using standard metabolic equivalent (MET) values [46]. Adult exercise/sports energy expenditure was estimated by the weighted average of energy expended in all activities reported over the five years preceding the interview (MET-h/week/year). For non-exercise activities, the authors estimated the energy expenditure in MET-h/week using appropriate MET values (i.e. walking: 3.3, cycling: 4, stair climbing: 9, and housework: 2) and the corresponding reported duration of the activity (hours/week).

Activities of 3 to 6 MET-h were classified as moderate and those >6 MET-h as vigorous intensity [46]. In order to evaluate adherence to CDC/ACSM guidelines, the authors calculated the proportion of women that met current recommendations of 150 minutes per week of moderate activity (e.g. Tai Chi, dancing, walking, cycling) or 60 minutes of vigorous activity (e.g. jogging, martial arts gymnastics, stair climbing) per week. Housework, categorized as light activity (2 MET-h), was not included in these calculations.

Occupational activity in the last job held by participants was categorized as low, medium, or high based on more than 300 different job titles using a coding system previously developed for the Shanghai urban population [47]. Clerks and accountants are examples of low activity occupations, while cooks and machine operators are examples of high activity occupations.

### Assessment of potential correlates of physical activity

Baseline information regarding demographics (age, marital status, family size (number of people living together), education, income, and employment history), lifestyle behaviors (cigarette smoking, alcohol consumption, tea consumption, ginseng intake, TV watching), menopausal status (defined as cessation of menstrual periods for 12 months or longer excluding lapses caused by pregnancy and breastfeeding), and medical history was collected through a self-administered questionnaire. Dietary data were collected by in-person interviews using a validated food frequency questionnaire [48] administered at the same time as the physical activity questionnaire. The food frequency questionnaire used in the SWHS includes 71 food items and food groups that cover about 86% of commonly consumed foods in urban Shanghai. For each food item or food group, subjects were asked how frequently (daily, weekly, monthly, yearly, or never) they consumed the food or food groups over the past year, followed by a question on the amount consumed in lians (50 g/lian) per unit of time. Daily energy intake and other nutrient intake data were derived from reported food intake and nutrient content listed in the Chinese Food Composition Tables [49]. Energy intake was included as a continuous variable in the current analysis.

Weight and height were measured by interviewers using a standardized protocol at the time of interview.

### Statistical analysis

The prevalence of various domains of physical activity was calculated as the percentage of women involved in that activity. Median, 25^th^, and 75^th ^percentiles are presented for continuous variables because their distributions were skewed. Body mass index (BMI) was calculated as weight (kg)/squared height (m^2^). BMI was categorized according to Chinese standards, that is: underweight to normal (<24 kg/m^2^), overweight (24.0–27.9 kg/m^2^), and obese (≥ 28 kg/m^2^) [50].

Correlations among energy expenditure estimates from various domains of physical activity were evaluated using the partial Spearman rank-correlation coefficient (r). For practical reasons the authors dichotomized energy expenditure (MET-h/week) from non-exercise activities at the median to create binary variables to be used in logistic regression. The associations of physical activity domains with demographics, behavioral factors, health status, and dietary energy intake were evaluated by odds ratios (OR) and 99% confidence intervals (CI) derived from logistic regression. Given the large sample size available for the analyses, the level of statistical significance was set at 0.01 and all tests were two-tailed. Statistical analyses were performed using SAS version 9.1 for Windows (SAS Institute, Carry, NC).

## Results

Demographic characteristics of the study population have been previously published [44]. In brief, the mean age of the participants was 52.1 years (standard deviation 9.1), and 50.4% of them were premenopausal. Most of the women reported education at the middle school level or below (58.5%); a large proportion had their longest job in the manufacturing sector (47.4%) and almost half (48.9%) were employed at baseline. About half of the women were of normal body weight and 12% were obese according to the Chinese classification of BMI.

### Prevalence and patterns of physical activity

Physical activity patterns reported during adolescence and adulthood are presented in table 1. More than three fourths of the women participated in exercise/sports during adolescence, with a median duration of two hours per week. This finding may reflect participation in physical education classes in middle and high school. The prevalence of reported regular exercise during the preceding five years was 35.5%, and 50% for women who reported exercising more than two hours per week. Those who exercised during adolescence were more likely to be adult exercisers (OR: 1.5, 99% CI: 1.4, 1.5). Most of the regular exercisers (76.4%) reported participation in only one activity, 19% in two activities and 5% in three activities (data not shown). The most common types of exercise reported were fitness-oriented traditional Chinese exercises of moderate intensity. The prevalence of vigorous activity reported was low (8% of exercisers). In contrast, active commuting to work (referred to as transportation), especially walking, was reported by more than 90% of women who were employed at the time of interview. Among women reporting these activities, the median duration of walking was about five hours per week and cycling to and from work about three hours per week. Almost all women reported walking, and only 8% reported biking for purposes other than transportation to work (referred to as daily activity). A large amount of time was spent in housework (median: 14 hours/week). Only 7% of participants reported recent jobs that required high levels of activity.

**Table 1:** Physical activity levels of middle-aged and elderly Chinese women, SWHS, 1997–2000

	**Number of participants (%)**	**Median (25^th^, 75^th ^percentiles)**
**I. Adolescence (13–19 years of age)**		
Exercise/sports	58,071 (77.5)	
Hours/week*		2 (2, 5)
Part of a sports team/Participation in tournaments	20,472 (27.3)	
		
**II. Adult**		
**Exercise/Sports (past 5 years)**	26,612 (35.5)	
Most common exercise types^†^		
Tai Chi and other slow movement exercises	21,107 (79.3)	
Dancing	3,760 (14.1)	
Walking	1,003 (3.8)	
Jogging	897 (3.4)	
Martial arts gymnastics	821 (3.1)	
		
Exercise intensity^‡^		
Moderate	24,595 (92.4)	
Vigorous	2,017 (7.6)	
		
Sweating during exercise		
Usually did not sweat	11,329 (42.6)	
Sweat most of the time	6,618 (24.9)	
Sweated every time	8,656 (32.5)	
		
Hours/week*		2.1 (1.1, 4.2)
		
MET-h/week*		*9.2 (4.6, 17.5)*
		
**Non-exercise activities (past year)**		
Stair climbing	61,186 (81.6)	
Number of flights of stairs/week*		84 (56, 12.6)
		
Transportation^§^		
Walking	36,225 (94.6)	
Hours/week*		5.4 (2.9, 12.5)
Cycling	13,711 (35.8)	
Hours/week*		3.4 (2.5, 5)
		
Daily activity^||^		
Walking	74,818 (99.8)	
Hours/week*		10.5 (7, 14)
Cycling	6,110 (8.2)	
Hours/week*		3.5 (1.8, 3.5)
		
Housework		
Amount done by participant		
Less than half	8,044 (10.7)	
Half	15,720 (21.0)	
All	51,176 (68.3)	
Hours/week*		14 (14, 21)
		
*MET-h/week from non-exercise activities*		*94.6 (70.4, 125.3)*
		
*MET-h/week – Total*		*100.2 (74.2, 131.5)*
		
**III. Occupational activity (last job)**		
Activity level		
Low	32,159 (43.1)	
Medium	37,421 (50.2)	
High	5,033 (6.8)	

*Calculated only for those involved in the corresponding activity (nonzero values)

^†^Each exercise type (up to three) reported by a person is counted in the corresponding category; therefore, the percentages may exceed 100

^‡^Women who participated in both vigorous (> 6 MET) and moderate (3–6 MET) activities are included in the vigorous activity category

^§^Active transportation to/from work; includes only women employed at the time of interview.

^||^Includes walking and cycling for daily errands (e.g. for shopping, etc)

Note: for different variables, the totals may vary because of missing values

When all moderate and vigorous non-occupational activities were taken into account, 63.8% of participants met current CDC/ACSM recommendations [51].

Overall, more than 90% of participants' reported physical activity energy expenditure was from non-exercise activities. Total energy expenditure was, on average, higher in regular exercisers than in non-exercisers (105.5 vs. 96.7 MET-h/week), but the amount of energy expended in non-exercise activities, most of moderate intensity, was higher in non-exercisers (96.7 vs. 92.1 MET-h/week) (table 2). Regular exercisers reported expending less energy in transportation and more energy walking and cycling for habitual daily activities than non-exercisers.

**Table 2:** Sources of physical activity energy expenditure, SWHS, 1997–2000

	**Non-exercisers**	**Exercisers**
	**MET-h/week** Median (25^th^, 75^th ^percentiles)	
Total	96.7 (70.9, 128.7)	105.5 (80.9, 135.8)
Exercise/sports		9.2 (4.6, 17.5)
Non-exercise activities	96.7 (70.9, 128.7)	92.1 (69.1, 119.9)
Transportation*	24.8 (14.9, 49.5)	23.8 (13.8, 41.3)
Daily activity^†^	34.7 (23.1, 46.2)	46.2 (23.1, 57.8)
Stair climbing	5.3 (2.1, 8.4)	5.3 (2.1, 8.4)
Household activities	28.0 (28.0, 42.0)	28.0 (28.0, 42.0)

*Transportation to/from work

^†^Includes walking and cycling for daily errands (e.g., for shopping, etc)

As presented in table 3, energy expenditure from exercise/sports was correlated inversely and weakly with that from transportation (r = -0.11); inverse correlations between transportation and daily activities (r = -0.24) and housework (r = -0.23), respectively, were also noted. A positive correlation was observed between energy expenditures in daily activities and housework (table 3).

**Table 3:** Age-adjusted Spearman correlation coefficients between domains of physical activity (MET-h/week), SWHS, 1997–2000

**Physical activity domain**	Exercise/sports	Transportation*	Daily activity^†^	Stair climbing	Housework
Exercise/sports	1.00				
Transportation	-0.11	1.00			
Daily activity^†^	0.08	-0.24	1.00		
Stair climbing	0.02	0.11	0.05	1.00	
Housework	0.03	-0.24	0.45	0.04	1.00

*Transportation to/from work

^†^Includes walking and cycling for daily errands (e.g., for shopping, etc)

### Correlates of exercise/sports and non-exercise activities

Older age and being unemployed (including retired and never employed) were positively associated with participation in exercise/sports and higher levels of housework (tables 4 and 5). Interestingly, some of the associations of physical activity domains with socio-demographic and lifestyle variables were in opposite directions for exercise/sports and non-exercise activities. For example, women with more education, more income, who were professionals, or had a smaller family size were more likely to report exercise participation, but they reported fewer non-exercise activities. Being a widow was not associated with participation in sports/exercise, but widows were more likely to walk or bike for transportation to and from work (OR: 1.26) and were less likely to do housework (OR: 0.87) than currently married women. Women who had never been married were less likely to exercise regularly or to expend energy in daily activities and housework. Chronic disease history was positively associated with exercise/sports (OR: 1.20) and somewhat inversely associated with non-exercise activities (ORs between 0.90 and 0.96). The most common types of exercise among women with a history of chronic disease were Tai Chi and other slow movement exercises (84.8%).

**Table 4:** Association of exercise/sports with socio-demographic and behavioral factors, health status, and energy intake, SWHS, 1997–2000

	**Sports/exercise**			
			
			Fully adjusted*	
			
	**No (n, %)** ** (n = 48,330)**	**Yes (n, %)** ** (n = 26,612)**	**OR**	**99% CI**
**I. Socio-demographics**				
**Age**				
40–44	17,105 (35.4)	3,813 (14.3)	1.00	Referent
45–49	11,399 (23.6)	4,050 (15.2)	1.40	1.33, 1.48
50–54	6,558 (13.6)	3,985 (15.0)	1.73	1.62, 1.85
55–59	3,958 (8.2)	3,670 (13.8)	2.11	1.94, 2.30
60–64	4,562 (9.4)	5,163 (19.4)	2.55	2.34, 2.78
65–70	4,748 (9.8)	5,931 (22.3)	2.82	2.57, 3.09
**BMI (kg/m^2^)**				
<24	26,700 (55.3)	13,083 (49.2)	1.00	Referent
24–27.9	16,125 (33.4)	9,783 (36.8)	1.00	0.96, 1.03
≥28	5,457 (11.3)	3,733 (14.0)	0.94	0.89, 0.99
**Menopausal status**				
Premenopausal	29,263 (60.6)	8,507 (32.0)	1.00	Referent
Postmenopausal	19,057 (39.4)	18,099 (68.0)	1.25	1.18, 1.32
**Marital status**				
Married	43,621 (90.3)	22,878 (86.0)	1.00	Referent
Widowed	2,799 (5.8)	2,801 (10.5)	1.02	0.95, 1.08
Separated/Divorced	1,445 (3.0)	740 (2.8)	1.02	0.93, 1.13
Never married	465 (1.0)	193 (0.7)	0.82	0.68, 0.98
**Education**				
None/Elementary	8,321 (17.2)	7,862 (29.6)	1.00	Referent
Middle school	19,928 (41.2)	7,755 (29.1)	1.05	0.99, 1.11
High school	14,029 (29.0)	6,861 (25.8)	1.28	1.20, 1.36
College and above	6,042 (12.5)	4,131 (15.5)	1.44	1.33, 1.55
**Occupation^†^**				
Manual workers	24,469 (50.8)	13,309 (50.2)	1.00	Referent
Clerical	10,529 (21.9)	4,964 (18.7)	1.01	0.97, 1.06
Professional	13,133 (27.3)	8,256 (31.1)	1.06	1.01, 1.11
**Employment status**				
Employed	29,126 (60.3)	9,173 (34.5)	1.00	Referent
Not employed	19,204 (39.7)	17,439 (65.5)	1.87	1.79, 1.95
**Family income**				
Low	13,038 (27.0)	7,773 (29.2)	1.00	Referent
Middle	18,870 (39.1)	10,263 (38.6)	1.12	1.07, 1.17
High	16,409 (34.0)	8,573 (32.2)	1.28	1.22, 1.34
**Family size**				
≥3	41,513 (85.9)	19,657 (73.9)	1.00	Referent
2	6,060 (12.5)	6,031 (22.7)	1.28	122, 1.34
1	757 (1.6)	924 (3.5)	1.59	1.41, 1.78
**Chronic disease^‡^**				
No	32,232 (66.7)	13,685 (51.4)	1.00	Referent
Yes	16,098 (33.3)	12,927 (48.6)	1.20	1.16, 1.24
**II. Behavioral factors**				
**Regular cigarette smoking**				
Never	47,029 (97.3)	25,800 (97.0)	1.00	Referent
Ever^§|^	1,301 (2.7)	812 (3.1)	0.75	0.68, 0.83
**Regular alcohol consumption**				
Never	47,356 (98.0)	25,908 (97.4)	1.00	Referent
Ever^|^	974 (2.0)	704 (2.7)	1.26	1.13, 1.40
**Regular tea consumption**				
Never	33,944 (70.2)	18,669 (70.2)	1.00	Referent
Ever^¶^	14,386 (29.8)	7,943 (29.9)	1.15	1.11, 1.19
**Regular ginseng intake**				
Never	36,491 (75.5)	16,237 (61.0)	1.00	Referent
Ever**	11,839 (24.5)	10,375 (39.0)	1.50	1.45, 1.55
**TV (hours/day)**				
≤ 2	14,274 (29.5)	7,856 (29.5)	1.00	Referent
3–4	23,473 (48.6)	13,060 (49.1)	1.00	0.96, 1.04
>4	10,582 (21.9)	5,693 (21.4)	0.93	0.89, 0.97
	Median (25^th^, 75^th ^percentile)			
**III. Total dietary energy intake (1,000 kcal/day)**	1,636 (1,404.8, 1,904.1)	1,652 (1,421.5, 1,916.8)	1.21	1.17, 1.26

* Adjusted for all variables presented in the table; OR: odds ratio, CI: confidence interval

^† ^Longest job

^‡ ^Chronic disease includes: coronary heart disease, hypertension, stroke, chronic bronchitis, asthma, tuberculosis, chronic hepatitis, chronic pancreatitis, diabetes mellitus and cancer

^§ ^Had smoked at least one cigarette per day for more than 6 months continuously

^||^Had consumed alcohol at least 3 times per week for more than 6 months continuously

^¶ ^Had consumed tea at least 3 times per week for more than 6 months continuously

**Had consumed ginseng at least 5 times per year during the past 3 years

**Table 5:** Associations of non-exercise domains with socio-demographic and behavioral factors, health status, and energy intake, SWHS, 1997–2000

	Fully adjusted OR and 99% CI*					
	
	**Transportation^†^**		**Daily activity^‡^**		**Housework**	
**I. Socio-demographic**						
**Age**						
40–44	1.00	Referent	1.00	Referent	1.00	Referent
45–49	0.95	0.91, 1.00	0.93	0.89, 0.98	1.00	0.96, 1.05
50–54	0.90	0.84, 0.97	1.05	0.99, 1.12	1.21	1.14, 1.29
55–59	0.90	0.80, 1.01	1.11	1.01, 1.21	1.35	1.24, 1.46
60–64	0.80	0.69, 0.92	1.07	0.98, 1.16	1.35	1.23, 1.47
65–70	0.55	0.45, 0.67	1.02	0.93, 1.11	1.16	1.06, 1.26
**BMI (kg/m^2^)**						
<24	1.00	Referent	1.00	Referent	1.00	Referent
24–27.9	1.08	1.04, 1.14	1.06	1.03, 1.10	1.05	1.02, 1.09
≥28	0.99	0.91, 1.07	1.07	1.01, 1.12	1.01	0.96, 1.06
**Menopausal status**						
Premenopausal	1.00	Referent	1.00	Referent	1.00	Referent
Postmenopausal	1.00	0.93, 1.07	1.02	0.96, 1.08	0.98	0.92, 1.04
**Marital status**						
Married	1.00	Referent	1.00	Referent	1.00	Referent
Widowed	1.26	1.10, 1.43	1.05	0.98, 1.12	0.87	0.82, 0.93
Separated/Divorced	1.14	1.01, 1.30	0.98	0.89, 1.08	0.91	0.82, 1.00
Never married	0.82	0.66, 1.03	0.70	0.58, 0.83	0.60	0.50, 0.72
**Education**						
None/Elementary	1.00	Referent	1.00	Referent	1.00	Referent
Middle school	0.63	0.56, 0.71	0.93	0.88, 0.98	1.21	1.15, 1.28
High school	0.48	0.43, 0.54	0.75	0.71, 0.80	0.96	0.90, 1.02
College and above	0.35	0.31, 0.40	0.53	0.49, 0.57	0.65	0.61, 0.70
**Occupation^§^**						
Manual workers	1.00	Referent	1.00	Referent	1.00	Referent
Clerical	1.13	1.08, 1.20	0.97	0.93, 1.01	0.91	0.87, 0.94
Professional	0.78	0.73, 0.82	0.79	0.75, 0.83	0.76	0.72, 0.79
**Employment status**						
Employed			1.00	Referent	1.00	Referent
Not employed			3.21	3.09, 3.34	2.97	2.86, 3.09
**Family income**						
Low	1.00	Referent	1.00	Referent	1.00	Referent
Middle	0.90	0.85, 0.95	0.94	0.90, 0.98	1.01	0.97, 1.05
High	0.75	0.71, 0.80	0.79	0.76, 0.83	0.93	0.89, 0.97
**Family size**						
≥3	1.00	Referent	1.00	Referent	1.00	Referent
2	0.88	0.81, 0.96	0.82	0.78, 0.86	0.58	0.56, 0.61
1	0.73	0.59, 0.91	0.67	0.60, 0.76	0.26	0.23, 0.30
**Chronic disease^||^**						
No	1.00	Referent	1.00	Referent	1.00	Referent
Yes	0.92	0.87, 0.96	0.96	0.93, 0.99	0.90	0.87, 0.93
**II. Behavioral factors**						
**Regular cigarette smoking**						
Never	1.00	Referent	1.00	Referent	1.00	Referent
Ever^¶^	1.05	0.87, 1.26	0.87	0.79, 0.96	0.83	0.75, 0.91
**Regular alcohol consumption**						
Never	1.00	Referent	1.00	Referent	1.00	Referent
Ever**	1.03	0.89, 1.19	0.99	0.89, 1.10	0.90	0.81, 1.00
**Regular tea consumption**						
Never	1.00	Referent	1.00	Referent	1.00	Referent
Ever^††^	0.94	0.90, 0.98	0.96	0.93, 1.00	0.92	0.89, 0.95
**Regular ginseng intake**						
Never	1.00	Referent	1.00	Referent	1.00	Referent
Ever^‡‡^	0.96	0.92, 1.01	0.95	0.91, 0.98	0.90	0.87, 0.93
**TV (hours/day)**						
≤ 2	1.00	Referent	1.00	Referent	1.00	Referent
3–4	0.98	0.93, 1.02	1.04	1.00, 1.07	1.07	1.03, 1.11
>4	1.02	0.96, 1.09	1.09	1.04, 1.14	1.11	1.06, 1.16
**III. Total dietary energy intake (1,000 kcal/day)**	1.31	1.25, 1.38	1.38	1.33, 1.50	1.28	1.23, 1.33

* Adjusted for all variables presented in the table; OR: odds ratio, CI: confidence interval

^† ^Transportation to/from work; analyses conducted only among those employed at the time of interview

^‡ ^Includes walking and cycling for daily errands (e.g. for shopping, etc)

^§^Longest job

^||^Chronic disease includes: coronary heart disease, hypertension, stroke, chronic bronchitis, asthma, tuberculosis, chronic hepatitis, chronic pancreatitis, diabetes mellitus, and cancer

^¶ ^Had smoked at least one cigarette per day for more than 6 months continuously

**Had consumed alcohol at least 3 times per week for more than 6 months continuously

^††^Had consumed tea at least 3 times per week for more than 6 months continuously

^‡‡^Had consumed ginseng at least 5 times per year during the past 3 years

Among behavioral factors, cigarette smoking was negatively associated with regular exercise (OR: 0.75, 99% CI: 0.68, 0.83), while alcohol consumption (OR: 1.26, 99% CI: 1.13, 1.40), tea consumption (OR: 1.15, 99% CI: 1.11, 1.19), and ginseng intake (1.50, 99% CI: 1.45, 1.55) were all positively associated with regular exercise. Watching TV more than four hours/day was negatively associated with participation in sports/exercise, but was positively associated with housework and walking or biking for daily activities. Both exercise and non-exercise activities were significantly associated with increased total dietary energy intake (ORs between 1.21 and 1.38). Food groups had a very weak correlation with energy expenditure; therefore, these data are not presented in the tables.

## Discussion

The present analysis provides valuable insights into patterns and correlates of physical activity among urban, middle-aged and older Chinese women. In general, the study population is physically active with 63.8% of the women meeting current CDC/ACSM recommendations for moderate and vigorous activity, not including housework. Of note is that regular participation in exercise/sports contributed only about 10% to the total non-occupational physical activity energy expenditure, while active transportation (especially walking) for daily activities and commuting and housework were the main contributors to total daily energy expenditure. This finding underscores the importance of designing instruments that take into account all physical activity domains, especially in women, who have been consistently shown to be less likely to participate in leisure-time physical activity [22,25-28], but spend more time in household activities [29,30,37]. Women who were physically active during adolescence were also more likely to engage in exercise/sports during adulthood. Therefore, promotion of physical activity at younger ages may be an effective approach to increasing participation in regular exercise during adulthood. As expected, traditional Chinese exercises were more common in this population of middle-aged and older women. Studies conducted in the United States have consistently shown walking to be the most common leisure-time physical activity among adults of all ages [28,33,37,52]. In our study walking was also the most frequent type of physical activity when walking for exercise, for active transportation to/from work, and for other daily activities were considered together (> 90%). Consistent with previous reports from other Chinese populations [35], active transportation was a major component of energy expenditure from non-occupational activities in this population residing in urban Shanghai. However, the proportion of people acquiring motor vehicles is increasing in China [42], and this would be expected to reduce active transportation in the future.

Overall, energy spent in transportation was somewhat inversely correlated with energy expenditure from exercise/sports, daily activity, and housework, most likely reflecting time constraints and different determinants for these domains of physical activity.

Most of the results from this study regarding correlates of exercise/sports are similar to previous research conducted in industrialized countries [31]. Nevertheless, existing data regarding domain-specific correlates of physical activity, useful for tailoring health promotion strategies, are still insufficient. Sternfeld and colleagues (1999) found that demographic and psychosocial correlates varied by physical activity domains (exercise/sports, active living, household/care giving, and occupational activities) in a random sample of women from the Northern California Kaiser Permanente Medical Care Program and underscored the importance of further investigation in this direction [32].

In this study, similar to previous reports, indicators of high socioeconomic status [34-36] and fewer family responsibilities [33,34] were positively associated with participation in exercise/sports. This suggests better access to and understanding of information regarding the health benefits of physical activity. Active transportation and housework were inversely related to these demographic variables.

Most previous studies conducted in the United States and Europe have found that participation in physical activity decreases with age [25,26,32]. However, in this study, age was positively associated with exercise/sports participation, which is consistent with other studies conducted in Asia [53,54]. This effect is mainly due to increased exercise participation after retirement, which occurs between the ages of 50 and 55 for most Chinese women. Another interesting finding in this population was the positive association between medical history of chronic disease and exercise/sports, most likely an effect of women seeking healthy behaviors following disease diagnosis. As expected, most of these women engaged in traditional Chinese exercises. These observations underscore the need for careful assessment of the history of chronic disease as a potential confounder or effect modifier in etiologic studies of physical activity.

The positive associations of non-smoking status, tea consumption, and ginseng intake with participation in exercise/sports but not in non-exercise activities suggest that regular exercisers are prone to engage in other healthy behaviors as well. Similar to previous reports [33,38], alcohol consumption was positively associated with participation in exercise/sports. However, smoking and alcohol consumption are uncommon (2.4%, and 1.9%, respectively) in this population [44].

### Strengths and limitations

This study is one of the largest and most comprehensive population-based surveys investigating patterns and correlates of physical activity, and the first such study conducted among urban Chinese women. The population-based design and a participation rate of more than 90% allowed a direct estimation of the prevalence of physical activity levels in Chinese women from Shanghai and also minimized selection bias. However, these results cannot be generalized to non-urban or younger women who were not included in the SWHS [44].

The physical activity questionnaire was interviewer-administered and provided a comprehensive picture of participants' physical activity patterns by collecting extensive and detailed information on the type, duration, and length of various domains of physical activity. A limitation of this questionnaire is that it did not distinguish between differences or changes in exercise habits that might have occurred during the 5-year period covered by the questionnaire and current exercise patterns. However, in the validation study of the PAQ, we found that the 5-year average of exercise habits correlated well with exercise during the preceding year as measured by multiple 7-day PAQ and 7-day logs [45], suggesting that exercise patterns in our study population are relatively stable and that the averaged exercise for the preceding 5 years reflects the participants' current exercise patterns. Because of the cross-sectional nature of data, the temporality of the observed associations cannot be determined and no causal inferences can be made. These limitations should be considered when interpreting the results.

## Conclusion

This study describes the unique pattern of physical activity, dominated by non-exercise, moderate intensity activities in urban, middle-aged and elderly Chinese women. It also shows that correlates of physical activity vary by domain in this population. These findings have public health implications in designing interventions to promote participation in physical activity. Follow-up of this cohort of women will allow the identification of trends in physical activity and prospective investigation of the effect of physical activity on various health outcomes.

## Abbreviations

ACSM- American College for Sports Medicine

BMI- Body Mass Index

CDC- Centers for Disease Control and Prevention

CI- Confidence Interval

MET- Metabolic Equivalents of Task

OR- Odds Ratio

SWHS- Shanghai Women's Health Study

WHO- World Health Organization

## Competing interests

The author(s) declare that they have no competing interests.

## Authors' contributions

ALJ performed the statistical analyses, interpreted the findings and drafted the manuscript. WW participated in the statistical analyses, result interpretation and manuscript preparation. YTG participated in study design, directed subjects' recruitment and data collection and participated in manuscript preparation. CEM participated in the statistical analyses, result interpretation and manuscript preparation. GY participated in study design, subjects' recruitment and data collection and manuscript preparation. HLL participated in subjects' recruitment and data collection. WZ obtained funding, designed the study, and participated in manuscript preparation. XOS contributed to study design and implementation, oversaw the study project, and participated in result interpretation and manuscript preparation.

All authors read and approved the submitted manuscript.

## Pre-publication history

The pre-publication history for this paper can be accessed here:


